# Preparation and investigation of nano-thick FTO/Ag/FTO multilayer transparent electrodes with high figure of merit

**DOI:** 10.1038/srep20399

**Published:** 2016-02-02

**Authors:** Shihui Yu, Lingxia Li, Xiaosong Lyu, Weifeng Zhang

**Affiliations:** 1 School of Electronic and Information Engineering, Tianjin University, Tianjin 300072, P. R. China; 2 Key Laboratory of Photovoltaic Materials of Henan Province and School of Physics and Electronics, Henan University, Kaifeng 475004, P. R. China

## Abstract

In order to improve the conductivity of the single–layered nano-thick F doped SnO_2_ (FTO) thin films, an Ag mid–layer is embedded between the FTO layers. In our work, the effects of mid–layer Ag and top FTO layer on the structural, electrical and optical properties of FTO/Ag/FTO multilayered composite structures deposited on quartz glass substrates by magnetron sputtering at 100 °C have been investigated. As the thickness of Ag mid–layer increases, the resistivity decreases. As the top FTO layer thickness increases, the resistivity increases. The highest value of figure of merit φ_TC_ is 7.8 × 10^−2 ^Ω^−1^ for the FTO (20 nm)/Ag (7 nm)/FTO (30 nm) multilayers, while the average optical transmittance is 95.5% in the visible range of wavelengths and the resistivity is 8.8 × 10^−5 ^Ω·cm. In addition, we also describe the influence of Ag and top FTO layer thickness on structural, electrical and optical properties of the nano-thick FTO (20 nm)/Ag/FTO multilayers and the mechanism of the changes of electrical and optical properties at different Ag and top FTO layer thicknesses.

Transparent conductive oxides (TCOs) are materials that exhibit high transmittance in the visible region along with high electrical conductivity^1^,^2^,^3^,^4^. Which have been studied and applied extensively as transparent electrodes for optoelectronic applications such as flat–panel displays, thin–film transistor liquid crystal displays, organic light emitting devices (OLED) and solar cells^5^,^6^,^7^,^8^,^9^. One of the most common TCOs is indium tin oxide (ITO), which offers commercially acceptable performance in terms of high conductivity and transparency^10^. A TCO needs to be cost effective, reliable and stable in order for it to be used as anode material in solar cells, *et al.*^11^,^12^,^13^ However, ITO thin films are expensive due to the high cost of indium. In addition, they are less stable in hydrogen plasma and are toxic^14^. Therefore, it is very important to study the properties of indium–free or indium–reduced TCO thin films for the application in solar cells. Among the possible materials, tin oxide is considered as one of the most promising candidates mainly because of its inexpensive cost, chemically stable in acidic and basic solutions, stable in hydrogen plasma, and mechanically strong, which are important attribute for the fabrication and operation of solar cells^15^,^16^,^17^. The undoped SnO_2_ thin films have high resistivity due to its stoichiometric nature which could not produce large number of free charge carriers. In order to increase the carrier density, the effective dopants have been used, such as antimony (Sb)^18^,^19^, niobium (Nb)^20^, tantalum (Ta)^21^, Cadmium (Cd)^22^, tungsten (W)^23^, Cobalt (Co)^24^, and fluorine (F)^25^,^26^,^27^. Among these dopants, F is found to be the most commonly used dopant for SnO_2_. F–doped SnO_2_ (FTO) films are wide bandgap semiconductor materials (Eg = 3.65 to 4.25 eV)^25^,^28^ resulting in the optical transmittance properties in the visible region^27^. However, its resistivity is still not low enough in some cases for improved practical applications. In order to improve the conductivity of transparent conducting films, it is an effective way to change the constructions.

Recently, a nano-thick TCO/metal/TCO multilayer system has been studied^28^,^29^,^30^,^31^,^32^,^33^. This structure has lower overall thickness than the single–layer TCO thin film, low sheet resistance and high transparence because the nano-thick TCO/metal/TCO multilayer system can suppress the reflection from the metal mid–layer and obtain a higher transparent effect. In addition, compare with the metal nanonets and carbon nanotubes, the adhesion of between TCO/metal/TCO multilayer system and substrate is stronger (the metal nanonets or carbon nanotubes is bonded to substrate through the strength of the weak key of the Van der Waals force, which makes them difficult to be practice applied). Amongst metals, Ag is a good candidate for such multilayer films because of its low resistivity. The optical and electrical properties of the multilayer stack depend considerably on the thickness and deposition conditions of the Ag mid–layer. The Ag mid–layer should be thin, uniform and continuous for high transmittance and low resistivity. In this article, a sputtering method was used to deposit nano-thick FTO/Ag/FTO multilayers by simultaneous radio frequency (RF) magnetron sputtering of FTO and direct current (DC) magnetron sputtering of Ag on quartz substrates at 100 °C. We investigated the structural, electrical, and optical properties of multilayers deposited at various thicknesses of Ag and top FTO layer.

## Results

### Effect of the Ag mid-layer thickness on the properties of nano-thick FTO/Ag/FTO multilayers

**Figure 1:**
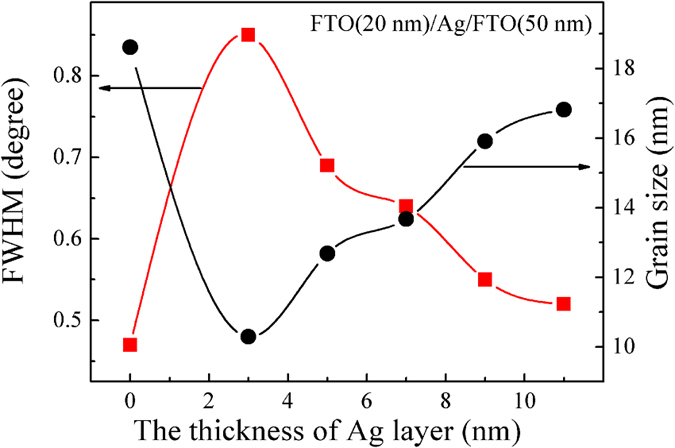
Grain size and FWHM of (2 1 1) peak of FTO (20 nm)/Ag/FTO (50 nm) multilayers prepared at various Ag mid–layer thickness.

The X–ray diffraction (XRD) results for sandwich structures FTO (20 nm)/Ag/FTO (50 nm) with different thicknesses of the middle Ag layer were shown in Fig. S1 of the Supplementary Information (SI). The change of crystal parameters references to the graph in Fig. S1. Figure 1 displays FWHM values of (2 1 1) diffraction peaks of FTO (20 nm)/Ag/FTO (50 nm) multilayers grown at various Ag mid–layer thickness at 100 °C. The value of FWHM decreases gradually with the increase of Ag mid–layer thickness. This also indicates that the variation of the Ag mid–layer morphology at increased Ag mid–layer thickness can enhance the crystal quality of top FTO thin film. In order to attain the detailed structure information, we calculated the grain size from the (2 1 1) orientation according to the Scherrer’s formula^34^,^35^.


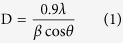


where D is the mean grain size, λ is the wave length of Cu Kα radiation (1.5418 Å ), β is the (2 1 1) peak width and θ is the Bragg diffraction angle. It is observed that the grain size increases from 10 nm to 17 nm with an increase in the Ag mid–layer thickness. That is to say, increasing the thickness of middle Ag layer enlarges the grain size of the top FTO layer. The change of surface morphology with the thickness of middle Ag layer references to the graph in Fig. S2 of SI.

Generally, the electrical conductivity of a semiconductor is determined by the concentration and Hall mobility of carriers as follows:


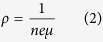


where ρ is the resistivity, *n* is the number of charge carriers, *e* is the charge of the carrier, and *μ* is the carrier mobility. The resistivity is inversely proportional to the carrier concentration and carrier mobility, closely related to the film structure.

**Figure 2:**
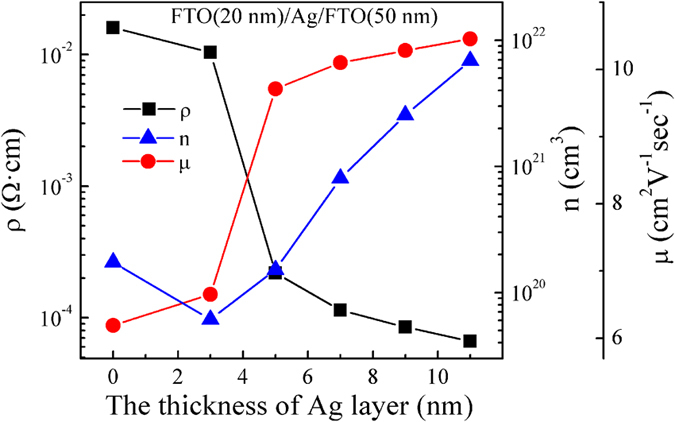
The dependence of electrical properties for FTO (20 nm)/Ag/FTO (50 nm) multilayers on the Ag mid–layer thickness.

Figure 2 shows the resistivity, mobility and carrier concentration of the FTO (20 nm)/Ag/FTO (50 nm) multilayers as a function of Ag mid–layer thickness. For the single–layered FTO thin film (70 nm) in this study, the resistivity is 1.61 × 10^−2 ^Ω·cm. After insertion of the 3 nm Ag mid–layer, the resistivity decreases to 1.03 × 10^−2 ^Ω·cm. As the Ag mid–layer thickness further increases to 11 nm, the resistivity decreases to the lowest value of 7.18 × 10^−6 ^Ω·cm for the FTO (20 nm)/Ag (11 nm)/FTO (50 nm) multilayers. The changes in resistivity between 0 nm and 7 nm Ag mid–layer can be attributed to the transition of Ag atoms from distinct islands to continuous thin film^36^. As the Ag mid–layer thickness increases further to 11 nm, the resistivity varies little implying that the conductivity of the Ag mid–layer tends to saturate. The decrease in resistivity can be known by inspection of the changes in carrier concentration and mobility. The mobility of multilayer thin film with 3 nm Ag mid–layer is lower than that of the single–layered FTO thin film. This suggests that most of the current passes through the FTO layer with the Ag islands acting as discontinuous scattering sites and further reduces the mobility. Besides, the poor crystallinity of FTO layer is also the reason for reducing the mobility. There are a large number of defects in the thin films with poor crystallinity, the mobility of the thin films is reduced due to the scattering from the defects (scattering centers)^37^,^38^. With the Ag mid–layer thickness increasing, the Ag mid–layer becomes continuous and the crystallinity of top FTO layer becomes better, and then the scattering decreases. As a consequence, both the mobility and carrier concentration of the multilayers increases with the increase of the Ag mid–layer thickness. The Ag mid–layer could inject the electrons into FTO thin film and reduce the resistivity of the FTO/Ag/FTO structure^33^, which can be understood based on the Schottky theory. Ag has a work function of *W*_*M*_ = ~4.26 eV^39^,^40^ and FTO has a work function of *W*_*S*_ = 4.9 ~ 5.0 eV^41^,^42^. As a consequence, there is formation of an Ohmic contact at the metal–oxide interface with many carrier electrons accumulated in the FTO layer. Owing to the difference of work function between Ag and FTO is large, there is significant injection of electrons into the FTO layer. To compare the carrier concentration of the single–layered FTO thin film with around 5.49 × 10^19 ^cm^−3^, the carrier concentration of FTO (20 nm)/Ag/FTO (50 nm) with 11 nm thick Ag mid–layer is increased by around three orders of magnitude to 1.03 × 10^22 ^cm^−3^. And, the carrier concentration get two orders of magnitude increase from 3–7 nm then another order of magnitude from 7–11 nm, suggesting that the Ag mid–layer becomes near–continuous when the thickness is 7 nm.

**Figure 3:**
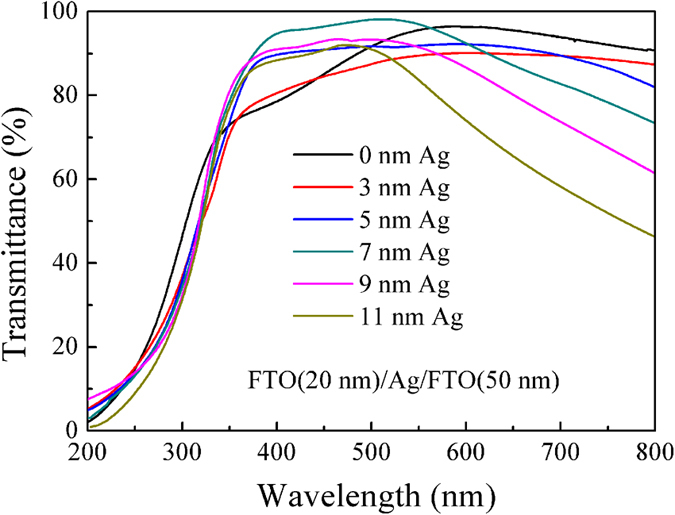
Transmittance spectra of FTO (20 nm)/Ag/FTO (50 nm) multilayers deposited at various Ag mid–layer thicknesses.

Figure 3 shows the transmission spectra in the wavelength range of 200–800 nm for the FTO (20 nm)/Ag/FTO (50 nm) multilayers on quartz substrate with different Ag mid–layer thicknesses deposited at 100 °C. The average optical transmittance Tav can be defined as follows^43^:


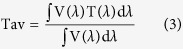


where V(λ) is the luminous spectral efficiency, and T(λ) is the measured transmittance of film system. The range of λ is from 380 nm to 780 nm. V(λ) approaches zero beyond this region and reaches the maximum of 1 at 555 nm (the medium of this region). From our calculation, the average optical transmittance of the single–layered FTO thin film is about 94.6% in the visible wavelength of 380–780 nm. Upon insertion of the 3 nm thick Ag mid–layer, the average optical transmittance drops to 89.1%. Further increasing the Ag mid–layer thickness to 7 nm, the average optical transmittance can reach to 95.9%. However, when the Ag mid–layer thickness is larger than 7 nm, the average transmittance decreases in the visible region with an increase of Ag mid–layer thickness. At a lower Ag mid–layer thickness (<7 nm), a fairly low average optical transmittance is observed due to the absorption and scattering of the aggregated Ag islands. Also, the decrease in average optical transmittance is due to scattering of light from the isolated Ag islands. Increasing Ag mid–layer thickness leads to the improvement of the average optical transmittance because the continuous Ag mid–layer has less scattering loss. However, as the Ag mid–layer thickness further increases, the average optical transmittance decreases due to higher plasmon absorption and light reflectance^30^.

In various applications of transparent conductive films, the optical and electrical properties of the films are very important. Ideally, both optical transmittance and electrical conduction should be as large as possible. However, their interrelation excludes the simultaneous achievement of maximum transmittance and conduction in most cases. We estimated a figure of merit *φ*_TC_ for the films defined as^43^


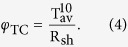


where *φ*_TC_ is the figure of merit, T_av_ is the transmittance (considering the application of the thin films to solar cells, we use the average transmittance) and R_sh_ is the sheet resistance (resistivity/thickness). This equation can be used to compare the performance of transparent conductive films. The figures of merit are 0.05 × 10^−2^, 0.02 × 10^−2^, 2.5 × 10^−2^, 6.8 × 10^−2^, 4.8 × 10^−2^ and 2.1 × 10^−2 ^Ω^−1^ for the 0, 3, 5, 7, 9, and 11 nm Ag mid–layers, respectively. The best figure of merit is obtained when the Ag mid–layer is 7 nm.

### Effect of the top FTO layer thickness on the properties of nano-thick FTO/Ag/FTO multilayers

According to the above results, we obtained that the multilayers exhibited the highest figure of merit φ_TC_ when the Ag mid–layer thickness is 7 nm. So the Ag mid–layer thickness was fixed at 7 nm as we investigated the properties of the multilayers deposited at various top FTO layer thicknesses. The changes of crystal parameters and surface morphology with the thickness of Ag mid–layer reference to the graph in Figs S3 and S4 of SI, respectively.

**Figure 4:**
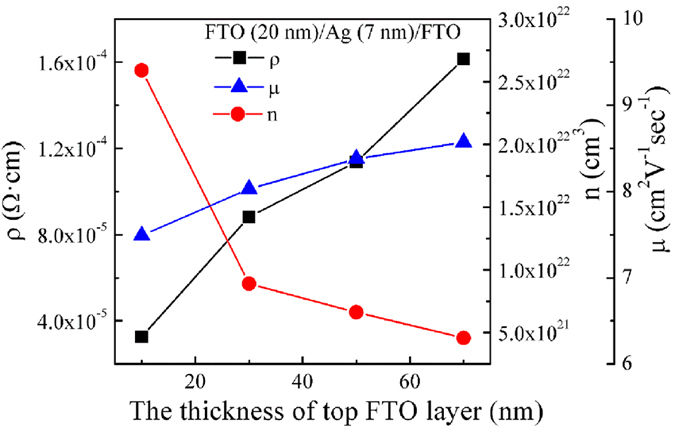
The dependence of electrical properties of FTO/Ag (7 nm)/FTO multilayers on the top FTO layer thickness.

Figure 4 presents the dependence of electrical properties of FTO (20 nm)/Ag (7 nm)/FTO multilayers deposited at 100 °C such as resistivity (ρ), carrier concentration (n), and Hall mobility (μ) on the thickness of the FTO layer. When the thickness of top FTO layer is 10 nm, the resistivity is 3.26 × 10^−5 ^Ω·cm. As we can see from Figure 4, the mobility values are observed to slightly increase with increasing top FTO layer thickness. The increase in resistivity is due to the changes in carrier concentration and mobility. The increased mobility is attributed to the improved crystallinity and increased crystallite sizes that weakens inter–crystallite boundary scattering and increases carrier lifetime^44^. However, as the top FTO layer thickness increases from 10 nm to 70 nm, the carrier concentration decrease slightly. The carrier concentration (*n*) can be evaluated using the following equation:


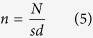


With


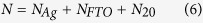



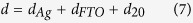


where *N* is the total number of carriers in the multilayers, *N*_*Ag*_ is the total number of carriers in the Ag mid–layer, *N*_*FTO*_ and *N*_*20*_ are the total number of carriers in the top FTO layer and bottom FTO layer, respectively. *s* is the surface area of multilayers. *d, d*_*Ag*_, *d*_*FTO*_ and *d*_*20*_ are the thickness of multilayers, Ag mid–layer, top FTO layer and bottom FTO layer, respectively. Since the total number of carriers of Ag mid–layer is much higher than FTO layer, a simpler relation can be used:


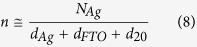


Then put the d_*Ag*_ = 7 nm and d_20_ = 20nm into the formula, Eq. (8) can be written as


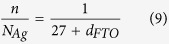


**Figure 5:**
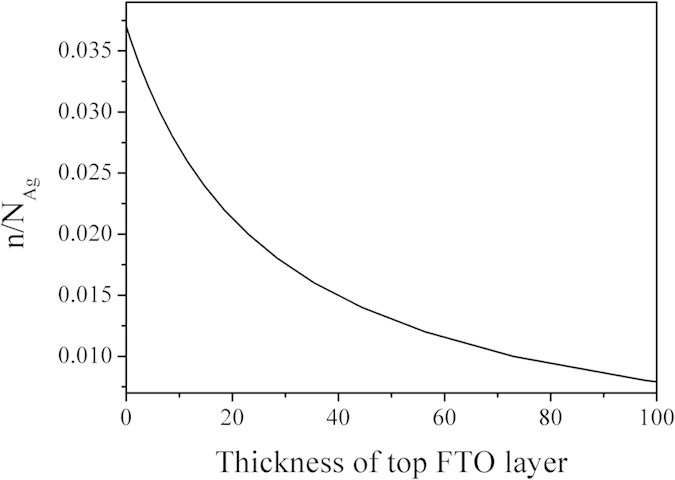
The thickness of top FTO layer versus *n/N*_*Ag*_.

Figure 5 shows the *d*_*FTO*_ dependence of *n/N*_*Ag*_. It can be clearly seen that the carrier concentration decreases as the increase of top FTO layer thickness. This consists well with the above experiment results.

**Figure 6:**
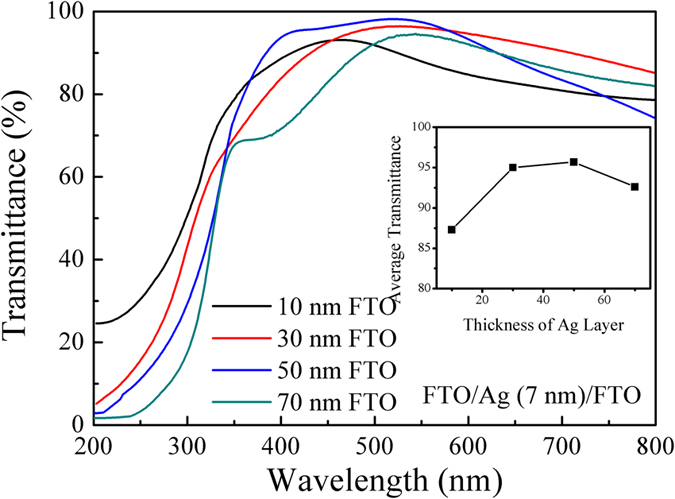
Transmittance spectra of FTO/Ag (7 nm)/FTO multilayers deposited at various top FTO layer thicknesses.

Figure 6 depicts the optical transmittance spectra of FTO (20 nm)/Ag (7 nm)/FTO multilayers with different top FTO layers thickness. It is observed in inset of Fig. 6 that the average optical transmittance in the visible range of wavelengths initially increases as the top FTO layer thickness increases, reaches a maximum (~96%) at the 50 nm thick top FTO layer, and slightly decreases with further increasing the top FTO thickness to 70 nm. The initial increase in average optical transmittance may be due to the decreased optical scattering caused by the decrease of defects in the top FTO layer owing to the improvement of crystallinity. However, as the top FTO layer thickness further increases, the average optical transmittance of the multilayers decreases due to the thickness effect^29^ (Thicker films tend to absorb more light and degrade optical transparency).

The figures of merit φ_TC_, which are determined according to Eq. (4), are 0.65 × 10^−2^, 7.8 × 10^−2^, 6.8 × 10^−2^, and 4.7 × 10^−3 ^Ω^−1^ for the 10, 30, 50, and 70 nm thick top FTO layers, respectively. The highest figure of merit (7.8 × 10^−2 ^Ω^−1^) is obtained when the Ag mid–layer is 7 nm and the top FTO layer is at 30 nm. The comparison of the best figure of merit between the literature and the proposed structures was summarized in Table 1. The highest figures of merit of nano-thick FTO/Ag/FTO multilayers prepared by magnetron sputtering in this paper is much higher than that of the other TCO/metal/TCO multilayer system, suggesting that nano-thick FTO/Ag/FTO multilayers have better optical and electrical properties, which is it is advantages for application in solar cells.

## Conclusion

In conclusion, nano-thick FTO/Ag/FTO multilayers were deposited on quartz glass substrates at 100 °C by by RF and DC magnetron sputtering. We investigated the structural, electrical, and optical properties of multilayer films deposited at various Ag mid–layer thicknesses and top FTO layers. As the Ag mid–layer thickness increases, the resistivity decreases. As the top FTO layer thickness increases, the resistivity increases. The lowest resistivity value of 7.18 × 10^−6 ^Ω·cm with a carrier concentration of 1.03 × 10^22 ^cm^−3^ was obtained at the optimum Ag (11 nm) and top FTO (50 nm) layer thickness. The highest value of figure of merit φ_TC_ is 7.8 × 10^−2 ^Ω^−1^ for the FTO (20 nm)/Ag (7 nm)/FTO (30 nm) multilayers, while the average optical transmittance is 95.5% in the visible range of wavelengths and the resistivity is 8.8 × 10^−5 ^Ω·cm. Which suggesting that FTO/Ag/FTO multilayers have good optical and electrical properties, it is advantages for application in solar cells.

## Methods

The nano-thick thin films of FTO and Ag were deposited on quartz glass substrates at 100 °C using a ceramic FTO and metallic Ag targets (99.99% purity, 5 cm diameter, 0.30 cm thickness) in an inline magnetron sputtering deposition system equipped with RF and DC power suppliers. The FTO target was mixed by SnO_2_ powder and SnF_2_ powder with the F concentration of 20 at.%. The mixed powder was pressed at 20 ton and sintered at 800 °C for 5 h in air to fabricated FTO ceramic target. The target–to–substrate distance was 6 cm. Prior to sputtering, the vacuum chamber was evacuated to a base pressure of lower than 5.0 × 10^−4 ^Pa. Thin film layer of FTO was deposited by RF magnetron sputtering onto quartz glass substrates at 50 w, the deposition rate was about 10 nm/min, high purity (99.999%) Ar (29 sccm) and O_2_ (1.0 sccm) were introduced into the chamber and controlled by mass flow meters with the total pressure maintained at 1.0 Pa. Ag mid–layer was deposited by DC magnetron sputtering at 30 w, the deposition rate was 0.5 nm/s, sputtering was performed at a pressure of 1 Pa in a pure Ar (30 sccm) atmosphere. The thickness of the bottom FTO layer was fixed at 20 nm (when the thickness is 20 nm, the contiguous FTO film without any holes just formed. When the thickness of FTO film above 20 nm, the thickness directly affects light transmission), and the thickness of the top FTO layer varied between 10 and 70 nm and the Ag mid–layer was varied between 0 and 11 nm. The thickness of FTO and Ag mid–layers was estimated based on the deposition time and deposition rate. The substrate temperature was measured using a thermocouple gauge and a hot cathode gauge. The variation of substrate temperature during deposition was maintained within ±1 °C.

### Characterization

X–ray diffraction (XRD) patterns were collected on a DX–2700 diffractometer with Cu Kα radiation (λ = 1.5418 Å). The surface morphologies were investigated by field emission scanning electron microscopy (FE-SEM, S-4800, Hitachi). The thickness of the thin films was measured by Alpha–Step D–100 profilometer (KLA–Tencor, California, USA). The electrical properties were measured by Hall measurements in the van der Pauw configuration (Ecopia HMS 3000 Hall System). Optical transmittance spectra and absorption spectra were obtained on an ultraviolet–visible–near infrared (UV–Vis–NIR) spectrophotometer (Varian Cary 5000) in the wavelength range 200–800 nm. All of the measurements were carried out at room temperature.

## Additional Information

**How to cite this article**: Yu, S. *et al.* Preparation and investigation of nano-thick FTO/Ag/FTO multilayer transparent electrodes with high figure of merit. *Sci. Rep.* **6**, 20399; doi: 10.1038/srep20399 (2016).

## Supplementary Material

Supplementary Information

## Figures and Tables

**Table 1:** Comparison of the best figure of merit between the literature and the proposed structures.

Ref.	Process method	Structure	Thickness (nm)	Highest figure of merit (×10^−2^ Ω^−1^)
45	Ion Beam Sputtering	ZnO/Ag/ZnO	(35/10/20)	1.6
33	Magnetron Sputtering.	ZnO/Cu/ZnO	(30/7/30)	0.87
46	Magnetron Sputtering	ITO/Ag/ITO	(40/10/40)	4.8
46	Magnetron Sputtering	ITO/Cu/ITO	(40/14/40)	0.4
47	Magnetron Sputtering	ZTO/Ag/ZTO	(20/8/39)	2.0
48	E–beam Evaporation	AZO/Ag/AZO	(30/12/30)	2.6
This study	Magnetron Sputtering	FTO/Ag/FTO	(20/7/30)	7.8
