# Supplementary material for: Preparation and investigation of nano-thick FTO/Ag/FTO multilayer transparent electrodes with high figure of merit
Source: Sci Rep. 2016 Feb 2;6:20399. doi: 10.1038/srep20399 (PMC4735849; doi:10.1038/srep20399)
Supplement: Supplementary Information [file srep20399-s1.doc]

**Preparation and investigation of nano-thick FTO/Ag/FTO multilayer transparent electrodes with high figure of merit**

**Shihui Yu^a^*, Lingxia Li^a†^, Xiaosong Lyu^a^, Weifeng Zhang^b^**

^a^School of Electronic and Information Engineering, Tianjin University, Tianjin 300072, P. R. China

^b^Key Laboratory of Photovoltaic Materials of Henan Province and School of Physics and Electronics, Henan University, Kaifeng 475004, P. R. China

Email:lingxiali@126.com, [ysh006@yeah.net](mailto:ysh006@yeah.net)

**Structural details of the FTO (20 nm)/Ag/FTO (50 nm) multilayers at different Ag layer thickness**

Figure S1 presents the X–ray diffraction (XRD) results for sandwich structures FTO (20 nm)/Ag/FTO (50 nm) with different thicknesses of the middle Ag layer, which ranged from 0 to 11 nm. The inset in Figure S1 illustrates the sandwich structure of FTO (20 nm)/Ag/FTO (50 nm) in the XRD study. FTO (20 nm)/Ag/FTO (50 nm) multilayers have two obvious tetragonal peaks of SnO_2_ corresponding to (1 1 0) and (2 1 1), respectively, and a weak Ag (1 1 1) peak. The absence of additional peaks in the XRD patterns excludes the possibility of any extra phases and/or large–size precipitates in the films. The FTO films show (2 1 1) preferred orientation. The similar XRD pattern behavior of the thin films can be found in the work reported by J. Montero group.^1^ Therefore, as the thickness of the Ag mid–layer increases, the intensity of Ag (1 1 1) increases, indicating the crystallinity of Ag is improved. A thick silver layer effectively enhanced crystal quality of the top FTO layer, as indicated by an increased FTO (2 1 1) diffraction intensity and a smaller full width at half maximum (FWHM).

In order to intuitive observation the surface morphology of FTO (20 nm)/Ag/FTO (50 nm) multilayers, the FE–SEM was used. Figure S2 shows the FE–SEM micrographs of FTO (20 nm)/Ag/FTO (50 nm) multilayers with different Ag mid–layer thickness. For the multilayers with the Ag thickness of 3 nm, the surface morphology is an incompact structure. When the Ag mid–layer is very thin, the Ag layer is discontinuous^2^, and the top FTO layer grows on the discontinuous Ag mid–layer, which results in the poor crystallinity, which is confirmed by XRD. When the Ag mid–layer thickness increases to 7 nm, and the surface morphology transforms to a compact and smooth structure implying the Ag mid–layer becomes continuous. The smooth structure can reduce the scattering of incident light, which makes some contribution to the increase of the transmittance^3^. For the film with the Ag mid–layer thickness of 11 nm, some grains are formed and uniformly distributed on the surface, which indicates that the crystallinity of the top FTO layer is improved distinctly.

**Structural details of the FTO/Ag (7 nm)/FTO multilayers at different top FTO layer thickness**

Figure S3 shows XRD patterns of the FTO (20 nm) /Ag (7 nm)/FTO multilayers deposited at different top FTO layer thicknesses. The XRD patterns exhibit that the structure of the multilayers is strongly dependent on FTO layer thickness. When the top FTO layer thickness is 10 nm, the multilayers show no characteristic XRD peaks except the Ag (1 1 1) peak indicating the amorphous nature of FTO thin film. With increasing the top FTO layer thickness to 30 nm, It is quite clear from Figure S3 that the top FTO layer with a poor polycrystalline structure that are oriented along the (1 1 0) and (2 1 1) planes. With a further increase of the top FTO layer thickness to 70 nm, the location of the measured diffraction peaks do not change significantly, but the intensities of the peaks increases. This is due to the crystallinity of the FTO thin film being improved and grain size becoming larger when elevating the film thickness^4^. The inset displays the FWHM values of (2 1 1) diffraction peaks of FTO (20 nm)/Ag (7 nm)/FTO multilayers deposited at different top FTO layer thicknesses. It is seen that the FWHM decreases from 2.02 to 0.53 as the top FTO layer thickness increases from 10 nm to 70 nm, indicating the increase of the average crystallite size due to the interaction and agglomeration with each other at thick FTO layer.

Figure S4 shows the surface morphology of the FTO (20 nm) /Ag (7 nm)/FTO multilayers deposited at different top FTO layer thicknesses. As shown in Figure 7, the surface morphology is strongly dependent on the thickness of top FTO layer. When the thickness of top FTO layer is 10 nm, the thin film is discontinuous and forms a collection of individual nanometer–sized clusters. With the increasing of top FTO layer thickness, the FTO layer become continuous, the average crystallite size of the film increased, and the crystallization of the film is enhanced.

**Supplementary references**

1. Montero, J., Herrero, J. & Guillén, C. Preparation of reactively sputtered Sb-doped SnO_2_ thin films: Structural, electrical and optical properties. *Sol. Energy Mater. Sol. Cells* **94**, 612–616 (2010).
2. Sivaramakrishnan, K. & Alforda, T. L. Metallic conductivity and the role of copper in ZnO/Cu/ZnO thin films for flexible electronics. *Appl. Phys. Lett.* **94**, 052104 (2009).
3. Wu, H. W., Yang, R. Y., Hsiung, C. M. & Chu, C. H. Influence of Ag thickness of aluminum-doped ZnO/Ag/aluminum-doped ZnO thin films. *Thin Solid Films* **520**, 7147–7152 (2012).
4. Marottia, R. E., Guerra, D. N., Bello, C., Machado, G. & Dalchiele, E. A. Bandgap energy tuning of electrochemically grown ZnO thin films by thickness and electrodeposition potential. *Sol. Energy Mater. Sol. Cells* **82**, 85–103 (2004). **Supplementary Figure S1**

**
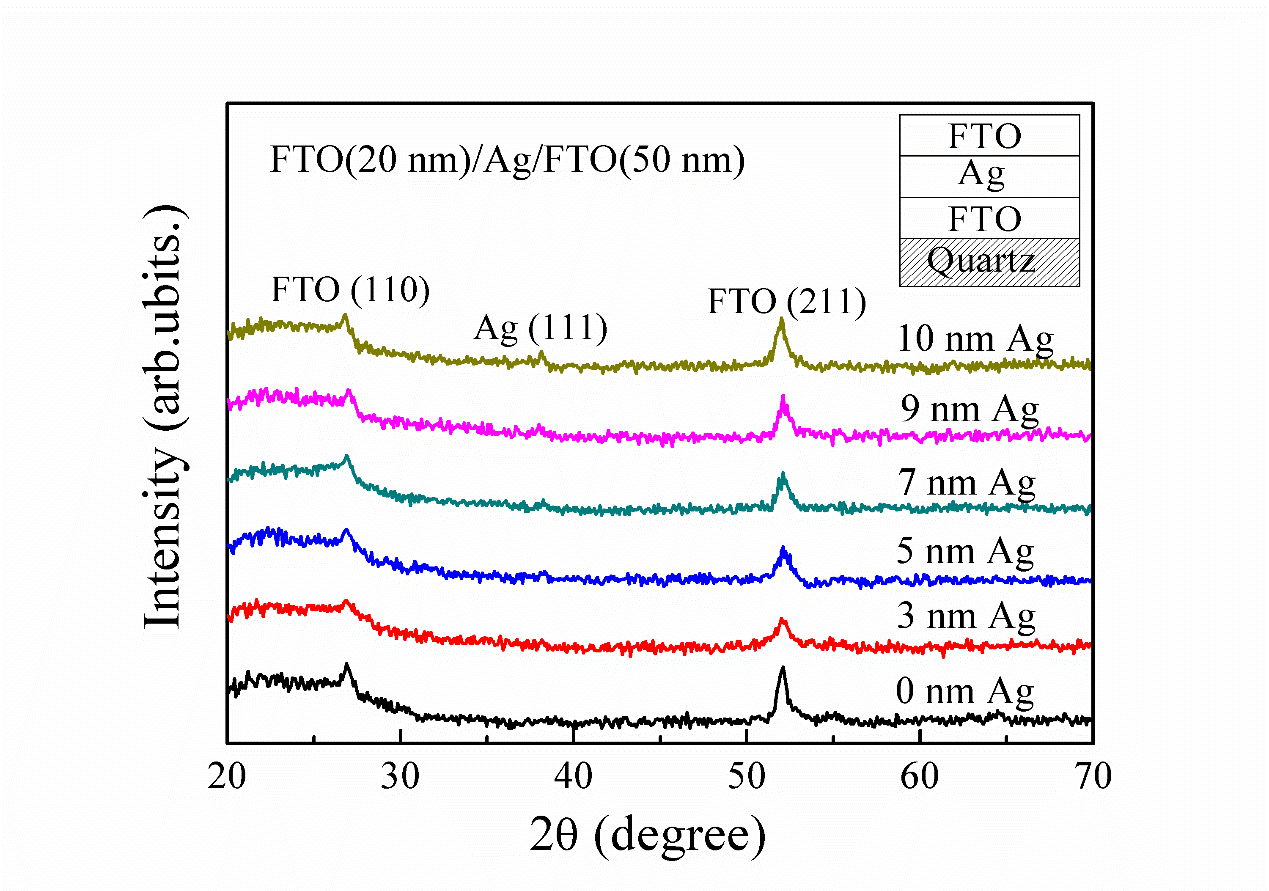
**

X–ray diffraction (θ–2θ) patterns as a function of Ag mid–layer thickness for FTO (20 nm)/Ag/FTO (50 nm) multilayers.

**Supplementary Figure S2**


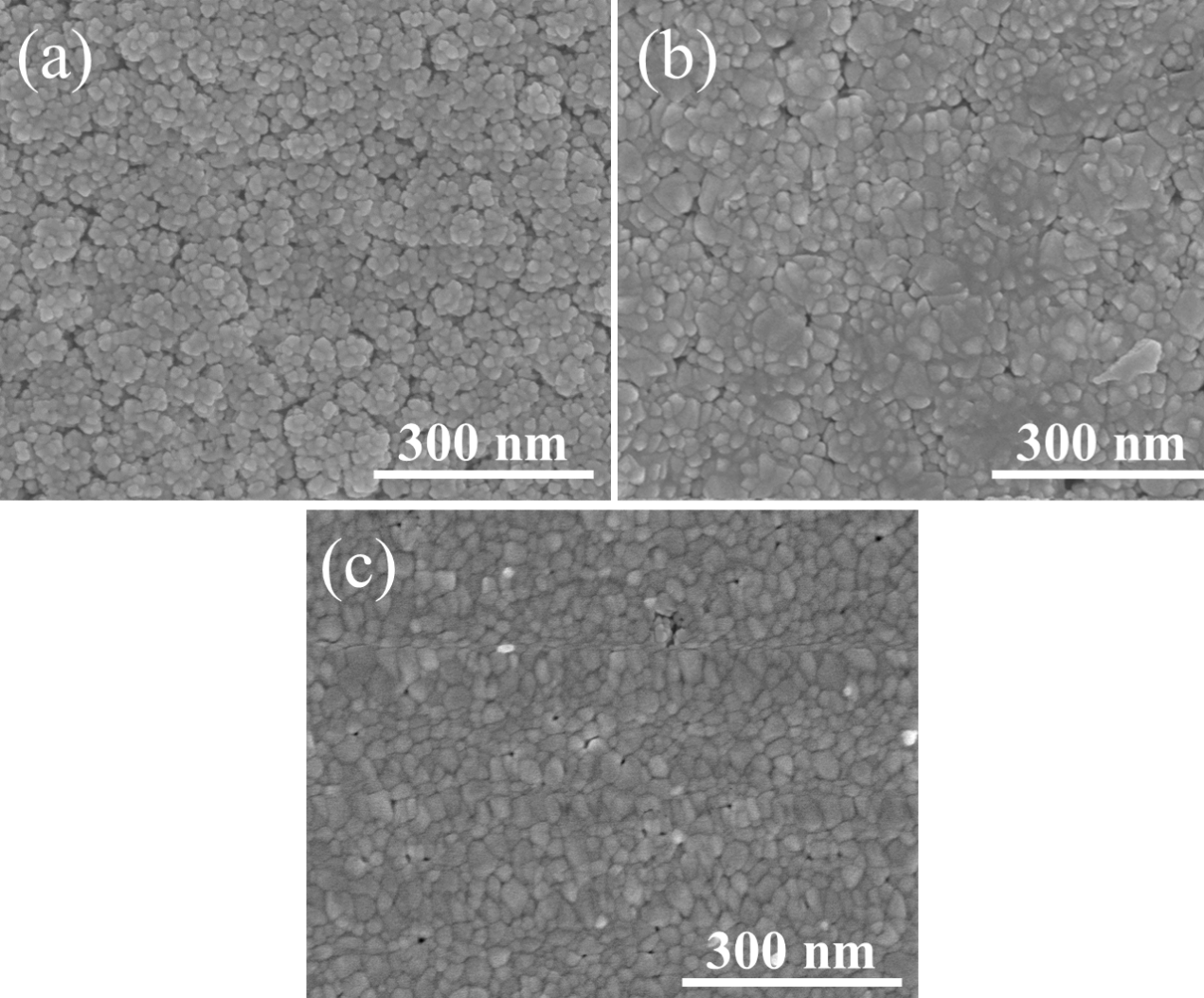


FE–SEM images of the FTO (20 nm)/Ag/FTO (50 nm) multilayers prepared at various Ag mid–layer thickness: (a) 3 nm, (b) 7 nm, (c) 11 nm.

**Supplementary Figure S3**


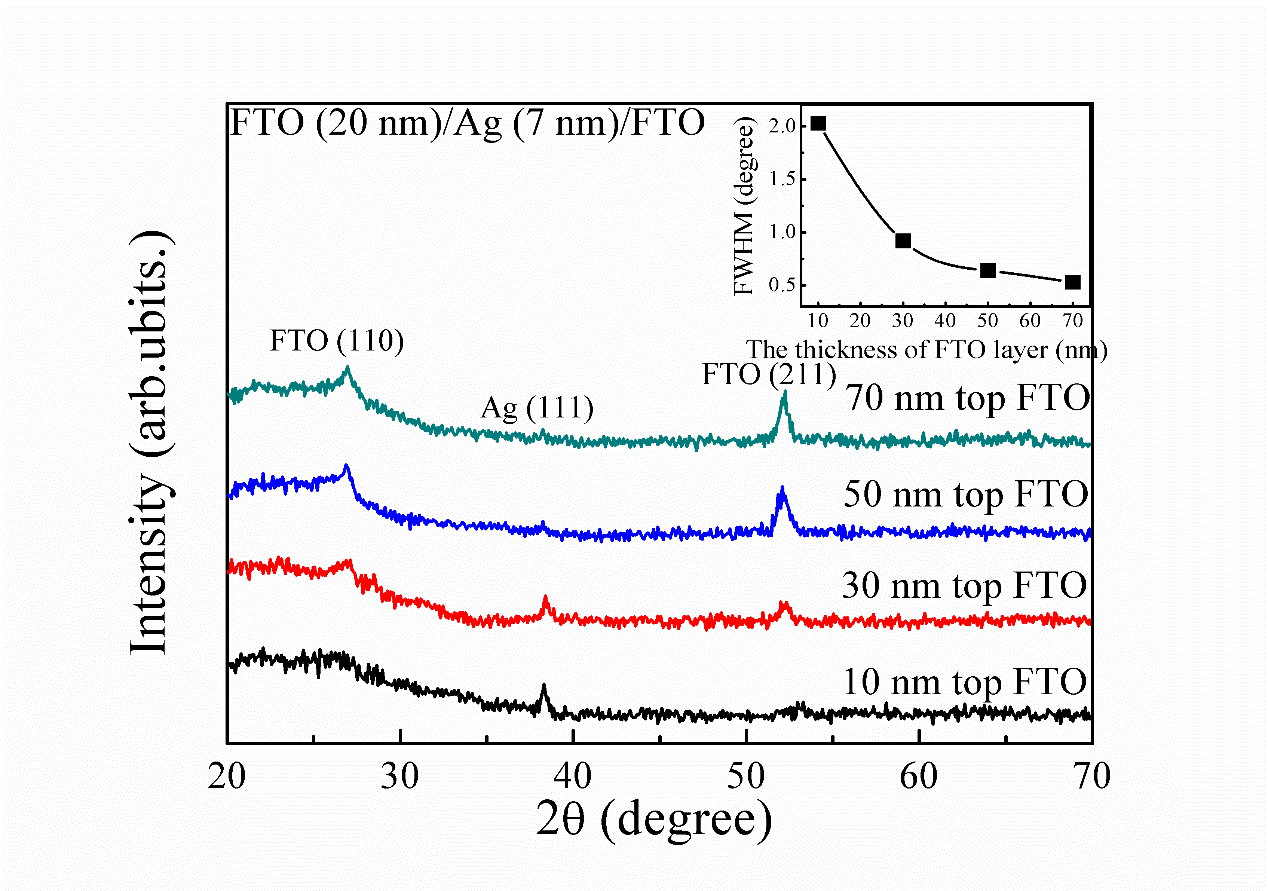


X–ray diffraction (θ–2θ) patterns of FTO/Ag (7 nm)/FTO multilayers at different top FTO layer thickness. The inset shows the FWHM value of (2 1 1) peaks of FTO/Ag (7 nm)/FTO multilayers at different top FTO layer thickness.

**Supplementary Figure S4**


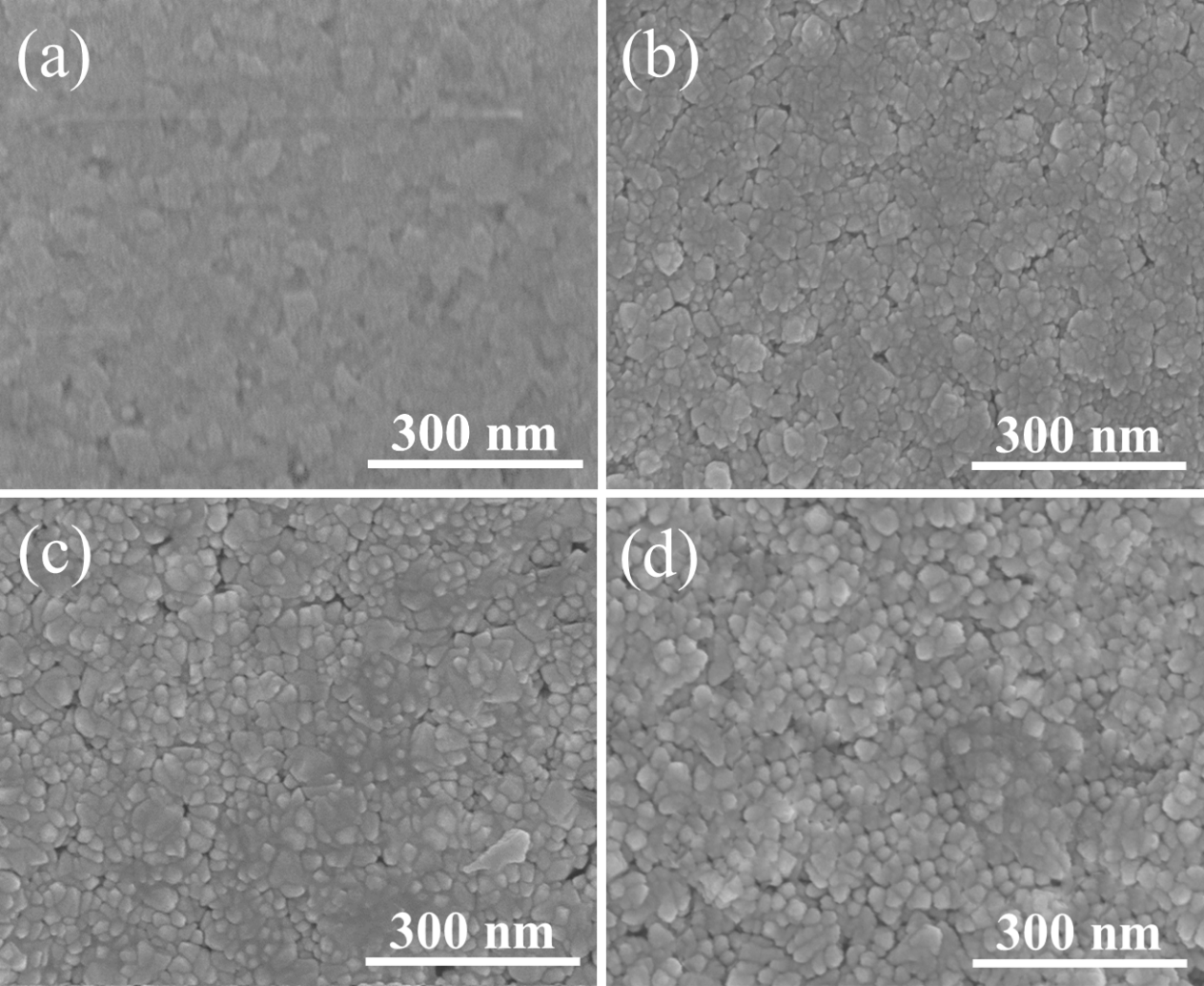


FE–SEM images of FTO/Ag (7 nm)/FTO multilayers different top FTO layer thicknesses: (a) 10 nm; (b) 30 nm; (c) 50 nm; (d) 70 nm.
